# Supplementary material for: Effects of population co-location reduction on cross-county transmission risk of COVID-19 in the United States
Source: Appl Netw Sci. 2021 Feb 18;6(1):14. doi: 10.1007/s41109-021-00361-y (PMC7891476; doi:10.1007/s41109-021-00361-y)
Supplement: Supplementary file 1 — Additional file 1. The results and visualization of statistical tests, metric values and cross-correlation analyses for the top 20 counties. [file 41109_2021_361_MOESM1_ESM.docx]

**Supplementary Information**

Effects of Population Co-location Reduction on Cross-county Transmission Risk of COVID-19 in the United States

Chao Fan^*^, Sanghyeon Lee, Yang Yang, Bora Oztekin, Qingchun Li, and Ali Mostafavi^*^

Chao Fan and Ali Mostafavi

Email: chfan@tamu.edu and mostafavi@tamu.edu

**This file includes:**

Table S1 – S3

Figure S1 – S3

**Table S1.** Number of counties in different week groups

|  | **High** | **Medium** | **Low** |
| --- | --- | --- | --- |
| **March 10** | 1009 | 1009 | 1009 |
| **March 17** | 1009 | 1009 | 1009 |
| **March 24** | 1009 | 1009 | 1009 |
| **March 31** | 1009 | 1009 | 1009 |

**Table S2.** P-values for statistical tests of county features among different county groups (county groups based on the CDC data on March 10, 17, 24, and 31 respectively)

|  | **March 10** | | **March 17** | | **March 24** | | **March 31** | | |
| --- | --- | --- | --- | --- | --- | --- | --- | --- | --- |
|  | **H-M** | **H-L** | **H-M** | **H-L** | **H-M** | **H-L** | | **H-M** | **H-L** |
| **Degree**  **Centrality** | 8.002e-29 | 7.260e-31 | 1.691e-67 | 2.590e-68 | 1.512e-73 | 6.95e-109 | | 1.330e-68 | 4.51e-123 |
| **Weekly New Cases** | 4.416e-07 | 4.106e-07 | 3.354e-80 | 1.750e-80 | 2.94e-297 | 0.0 | | 0.0 | 0.0 |
| **Population**  **Size** | 8.952e-15 | 5.889e-11 | 1.516e-80 | 4.055e-83 | 6.88e-180 | 2.34e-230 | | 2.01e-193 | 6.23e-275 |

**Note:** H means high level, M means medium level, and L means low level.

**Table S3.** P-values for the statistical tests of co-location reduction among different types of edges (county groups based on CDC data on March 10 and March 17 respectively)

|  | **HH-HM** | **HH-HL** | **MM-HM** | **MM-ML** | **LL-HL** | **LL-ML** |
| --- | --- | --- | --- | --- | --- | --- |
| **March 10** | 2.657e-44 | 2.727e-41 | 0.3973 | 0.0072 | 0.1897 | 0.0017 |
| **March 17** | 1.711e-53 | 2.639e-63 | 0.2915 | 0.0022 | 3.802e-07 | 0.7236 |

**Note:** HH means the edge connecting two counties in high-level group; HM means the edge connecting two counties in high-level group and medium-level group respectively; HL means the edge connecting two counties in high-level group and low-level group; so on and so forth.

**Fig. S1.** The heatmaps of aggregated edge weights (co-location probabilities) for different types of edges using different grouping criteria on different weeks. The heatmaps on the same raw are generated from the county groups categorized using CDC data on the same week, but the edge weights are based on the data from different weeks. The heatmaps on the same column are generated using the edge weights on the same weeks, but the counties are grouped by the CDC data on different weeks.

**Fig. S2-1.** Five metrics for top 10 counties across the studied weeks.

**Fig. S2-2.** Five metrics for top 11-20 counties across the studied weeks.

**Fig. S3.** Results of cross-correlation for co-location degree centrality and weekly basic reproduction numbers for top 20 counties.
